# Fibrin-Modified Cellulose as a Promising Dressing for Accelerated Wound Healing

**DOI:** 10.3390/ma11112314

**Published:** 2018-11-17

**Authors:** Marketa Bacakova, Julia Pajorova, Tomas Sopuch, Lucie Bacakova

**Affiliations:** 1 Department of Biomaterials and Tissue Engineering, Institute of Physiology of the Czech Academy of Sciences, 14220 Prague, Czech Republic; julia.pajorova@fgu.cas.cz (J.P.); lucie.bacakova@fgu.cas.cz (L.B.); 2 Holzbecher, Ltd.-Bleaching & Dyeing Plant in Zlic, 55203 Zlic, Czech Republic; tomas.sopuch@holzbecher.net

**Keywords:** fibrin, sodium carboxymethylcellulose, wound dressing, wound healing, dermal fibroblasts, skin

## Abstract

Dermal injuries and chronic wounds usually regenerate with scar formation. Successful treatment without scarring might be achieved by pre-seeding a wound dressing with cells. We aimed to prepare a wound dressing fabricated from sodium carboxymethylcellulose (Hcel^®^ NaT), combined with fibrin and seeded with dermal fibroblasts in vitro. We fabricated the Hcel^®^ NaT in a porous and homogeneous form (P form and H form, respectively) differing in structural morphology and in the degree of substitution of hydroxyl groups. Each form of Hcel^®^ NaT was functionalized with two morphologically different fibrin structures to improve cell adhesion and proliferation, estimated by an MTS assay. Fibrin functionalization of the Hcel^®^ NaT strongly enhanced colonization of the material with human dermal fibroblasts. Moreover, the type of fibrin structures influenced the ability of the cells to adhere to the material and proliferate on it. The fibrin mesh filling the void spaces between cellulose fibers better supported cell attachment and subsequent proliferation than the fibrin coating, which only enwrapped individual cellulose fibers. On the fibrin mesh, the cell proliferation activity on day 3 was higher on the H form than on the P form of Hcel^®^ NaT, while on the fibrin coating, the cell proliferation on day 7 was higher on the P form. The Hcel^®^ NaT wound dressing functionalized with fibrin, especially when in the form of a mesh, can accelerate wound healing by supporting fibroblast adhesion and proliferation.

## 1. Introduction

Cellulose-based materials have been widely applied in clinical practice as a wound dressing for treating acute and chronic wounds (for a review, see [1]). Cellulose is a biopolymer suitable for medical use due to its biocompatibility, non-cytotoxicity, low cost and good availability [2]. Cellulose can be modified into several forms, e.g., regenerated cellulose, oxidized cellulose, acetate cellulose, methylcellulose, hydroxypropylcellulose, carboxymethylcellulose and others, which differ in physicochemical properties [3,4]. Carboxymethylcellulose is prepared by carboxymethylation of any of the three hydroxyl groups in the glucose molecule. The average number of substituted hydroxyl groups is referred to as the degree of substitution. The degree of substitution influences the ability of the material to absorb water and to form a hydrogel. In addition, in the body, the degree of substitution and the molecular weight affect the absorbability of the material. The higher the degree of substitution of the material, the greater the amount of water that the material can absorb, and the longer the time that the water absorption takes [5]. Carboxymethylcellulose, or carboxymethylcellulose in the form of a sodium salt, successfully supports wound healing, prevents infection and promotes homeostasis [5,6,7].

Although a wide range of tools are available for wound treatment nowadays, scientists are still aiming to achieve faster and less painful healing with better aesthetic outcomes. In clinical practice, carboxymethylcellulose wound dressings are applied only to cover a healing wound, without seeding of the skin cells. However, successful healing is ensured by the cells migrating from the wound edge, producing specific growth factors and synthesizing extracellular matrix (ECM) proteins [8,9]. In the case of deep wounds, the natural healing process can be limited. The result may be a chronic non-healing wound, or extensive scar formation. Pre-seeding the wound dressing with skin cells can contribute to successful healing [10,11,12,13]. The colonization of a scaffold with cells depends strongly on the physical and chemical properties of the materials. In their pristine state, many synthetic and natural polymers used as carriers of skin cells fail to provide sufficient support for cell adhesion, growth and ECM deposition [14]. These materials can be combined with biomolecules physiologically present in the natural skin tissue (e.g., collagen, fibronectin), or occurring during wound healing (fibrin), in order to enhance colonization of the material with cells.

Fibrin is a protein resulting from the coagulation cascade. Blood plasma contains fibrinogen, a soluble protein which is converted to insoluble protein fibrin after an injury. This conversion is catalyzed by the enzyme thrombin. Fibrin forms a network in which the blood platelets and immune cells are trapped, and they form a blood clot together [14]. By producing growth factors (mainly platelet-derived growth factor), the platelets stimulate the fibroblasts to migrate into the wound, to proliferate and to form a new ECM composed mainly of collagen I and fibronectin [15,16]. In current clinical practice, fibrin is widely used to support wound healing in the form of a glue, a gel or a sealant seeded by dermal fibroblasts [16]. Promisingly, fibrin can also be used in the form of micro- and nanostructures. Micro- or nanostructured scaffolds mimic better the natural matrix environment for cell life. However, a fibrin scaffold as a self-supporting matrix for cells has weak mechanical strength and a fast rate of degradation. The mechanical stability of a fibrin scaffold can be improved by combining fibrin with other natural or synthetic polymers [17]. In our previous studies, we deposited fibrin on degradable polylactide nanofibers, and we observed improved adhesion and proliferation of dermal fibroblasts and enhanced synthesis of ECM proteins by these cells [18,19,20].

The aim of this study is to improve a clinically used carboxymethylcellulose wound dressing (Hcel^®^ NaT) by coating with fibrin and pre-seeding with dermal fibroblasts in order to create a cell carrier with potential to deliver skin cells into skin wounds. This novel cell-enriched wound dressing is expected to improve the healing ability of deep wounds.

The Hcel^®^ NaT was prepared in two forms that differed in structural morphology and degree of substitution. We functionalized these materials with fibrin of two different structures. The first fibrin structure, referred to as the fibrin mesh, coated the material fibers and formed a fine mesh among the fibers. The second structure, referred to as the fibrin coating, only coated the cellulose fibers. The purpose of our study was to investigate the effect of Hcel^®^ NaT morphology on cell proliferation and on the preparation of the fibrin structures, and also to evaluate the effect of fibrin and its structures on the adhesion and growth of primary human dermal fibroblasts. We supposed that the properties of Hcel^®^ NaT would influence the formation of the fibrin structures on the material and the behavior of dermal fibroblasts. We further expected an enhancing effect of fibrin functionalization on cell adhesion and proliferation. Moreover, we hypothesized that different morphology of fibrin structures would result in distinct cell behavior.

## 2. Materials and Methods

### 2.1. Material Preparation

A nonwoven textile made of sodium carboxymethylcellulose was prepared at the Holzbecher Bleaching & Dyeing Plant in Zlic (Zlic, Czech). This material is a commercially available product known as Hcel^®^ NaT. The highly pure cotton product PurCotton^®^ in a hydroentangled (i.e., spunlaced) textile structure was carboxymethylated and converted to a sodium salt. Two nonwoven textile forms with different structural morphologies and properties (molecular weight, degree of hydroxyl group substitution (DS) and pH of the aqueous extract) were prepared, namely the P form, containing visible pores, and the homogeneous H form, without visible pores. The textiles were sterilized by 25 kGy of γ-radiation and were packed in sterile Tyvek^®^-PE packaging (Wipak, Lahti, Finland). The samples were fixed into CellCrowns™ inserts (Scaffdex Ltd., Tampere, Finland) in order to prevent the sample floating in the cell culture medium during cell cultivation. 

### 2.2. Characterization of the Properties of Hcel^®^ NaT

The molecular weight of the P and H forms of Hcel^®^ NaT was determined by size exclusion chromatography (SEC). Sodium carboxymethylcellulose separation was performed in an asymmetric-flow AF4 system (Eclipse AF4, Wyatt Technology, Santa Barbara, CA, USA), using Long Channel (LC, 275 mm), a 350-µm spacer, and a 30 kg.mol^−1^ Regenerated Cellulose (RC) semipermeable membrane. The online concentration was detected by an RI detector (Optiplab T-rEX, Wyatt Technology, Santa Barbara, CA, USA). Angular dependent light scattering was detected by the MALS instrument (Dawn Heleos II, Wyatt Technology, Santa Barbara, CA, USA). The molar mass, the RMS radius (Rg) and other operations were calculated from the scattering and concentration data with the use of Astra software (version 6.1, Wyatt Technology, Santa Barbara, CA, USA). Berry formalism was used for the molar mass and RMS radius calculations. 100 µL of 3g/L sodium carboxymethylcellulose in 100 mmol/L NaCl was injected per single AF4 run. Dn/dc = 0.159 was used, in accordance with Melander and Vuorinen [21].

The degree of substitution was determined by titration of the sodium carboxymethylcellulose solution with an NaOH solution to pH change, in accordance with the European Pharmacopoeia and the American Pharmacopoeia.

To determine the pH of aqueous extracts of two forms of Hcel^®^ NaT, 1 g of the material was shaken in 100 mL of deionized water for 5 min. Then the pH of the Hcel^®^ NaT extract was measured. 

The morphology of the P and H forms of Hcel^®^ NaT was studied by scanning electron microscopy (SEM). The Hcel^®^ NaT fibers were sputter-coated with gold in argon background gas. Images were captured by a Quanta 450 scanning electron microscope (SEM) (FEI, Hillsboro, OR, USA) in a high vacuum (10^−4^ Pa). Detection was mediated by an Everhart-Thornley detector in secondary electrons mode in an accelerating voltage of 30 kV. The fiber width and the fiber thickness, and also the size of the void spaces among the fibers, were measured on SEM images of Hcel^®^ NaT of the P form and the H form in the ImageJ Fiji program. The fiber width and thickness data were presented as the arithmetic mean ±standard deviation (S.D.) in µm. The area of the void spaces among the fibers was expressed as the range from the minimum value to the maximum value in µm^2^. The data were calculated from 24 measurements per SEM image of the P form or H form.

### 2.3. Hcel^®^ NaT Functionalization with Fibrin

Two different structures of fibrin modifications (fibrin mesh and fibrin coating) were prepared in this study. The fibrin mesh formed a coating around the cellulose fibers, and additionally a fine mesh among the cellulose fibers. The fibrin coating covered only the cellulose fibers. The principle for preparing the fibrin structures is based on the physiological process of hemocoagulation, when soluble fibrinogen is converted by thrombin to insoluble fibrin [22]. The two fibrin structures, fibrin coating and fibrin mesh, were prepared in accordance with our previously published work [20]. Fibrinogen (EMD Millipore, Billerica, MA, USA, Cat. No. 341576) was dissolved in a Tris Buffer (consisting of 50 mM Tris-HCl, 100 nM NaCl and 2.5 mM CaCl_2_) to a final concentration of 10 µg/mL and it was left for 1 h to adsorb on the Hcel^®^ NaT fibers. The Hcel^®^ NaT was rinsed with Tris Buffer. Then thrombin (Sigma-Aldrich Co, St Louis, MO, USA, Cat. No. T6884) with a final concentration of 2.5 U/mL in Tris Buffer was added for 15 min. In the case of the fibrin coating, the samples were rinsed properly in Tris Buffer for 30 min. After rinsing, a solution of 200 µg/mL of fibrinogen in Tris Buffer and 0.5 U/mL of antithrombin III (Chromogenix, Milano, Italy) in deionized water was added to the Hcel^®^ NaT fibers for 1 h. In the case of the fibrin mesh, the solution of fibrinogen and antithrombin III was added to the samples immediately after thrombin catalysis, without the rinsing step in Tris Buffer. Finally, the samples were rinsed with Tris Buffer and with phosphate-buffered saline (PBS, Sigma-Aldrich Co., St. Louis, MO, USA).

### 2.4. Cell Culture Conditions

The Hcel^®^ NaT samples were seeded with neonatal human dermal fibroblasts (Lonza Group, Basel, Switzerland, Cat. No. CC-2509) in passage 3–5 at a concentration of 15,000 cells/cm^2^. The cells were cultured in 1.5 mL of Dulbecco’s Modified Eagle’s Medium (DMEM; Sigma-Aldrich, USA), supplemented with 10% of fetal bovine serum (FBS; Sebak, Aidenbach, Germany) and 40 µg/mL of gentamicin (LEK, Ljubljana, Slovenia). The cultivation was performed at 37 °C in an air atmosphere with 5% of CO_2_ and 90% humidity for 3 or 7 days.

### 2.5. Hcel^®^ NaT Cytotoxicity

The potential cytotoxicity of the Hcel^®^ NaT was tested by evaluating the growth of human dermal fibroblasts in extracts from the tested materials, using the xCELLigence^®^ real-time cell proliferation monitoring system (Roche, Switzerland). The extracts of Hcel^®^ NaT were prepared by sample incubation in DMEM without FBS and without seeded cells at 37 °C in an air atmosphere with 5% of CO_2_ and 90% humidity for 7 days. Effective extraction was achieved with the use of an SSM1 Mini Orbital Shaker (Stuart; speed 45 rpm). The cells at a concentration of 3000 cells/well in DMEM with 10% of FBS were seeded in a 10 µL suspension into a 96-well E-plate View (ACEA Biosciences, San Diego, USA). After the background impedance had been measured, 180 µL of the sample extract with 10% of FBS was added to the seeded cells. The cell proliferation was measured as the electrical impedance for 168 h with 8-h intervals. 

The pure culture medium was used as a control. The real-time proliferation measurement was performed under the same conditions as for cultivation of the cell on Hcel^®^ NaT. The arithmetic mean ± S.D. was determined from four parallel samples for each type of material and time point.

### 2.6. Morphology of the Fibrin Structures

The morphology of the fibrin mesh and the fibrin coating was observed on freshly prepared samples, and on day 3 and 7 after cell seeding. The fibrin structures were treated with 1% albumin in 0.1% Triton X-100 (Sigma-Aldrich Co., USA) for 15 min, and then with 1% Tween (Sigma-Aldrich Co., USA) for 15 min to block non-specific binding sites. After being rinsed twice with PBS, the fibrin was immunolabeled with primary polyclonal rabbit antibody against fibrinogen (Dako Denmark A/S, Glostrup, Denmark) diluted in PBS in a ratio of 1:200 for 1 h at 37 °C. Then the samples were rinsed twice with PBS and were incubated with a secondary antibody, i.e., goat anti-rabbit F(ab′)2 fragments of IgG (H + L) conjugated with Alexa Fluor^®^ 488 (Molecular Probes, Eugene, OR, USA), diluted in PBS in a ratio of 1:400, for 1 h in the dark. After rinsing with PBS, images of the morphology fibrin structures were taken under a Leica TCS SPE DM2500 confocal microscope (Leica Microsystems, Wetzlar, Germany; obj. 20×/0.70 NA oil, 40×/1.15 NA oil).

### 2.7. Cell Adhesion and Proliferation

We evaluated the cell attachment, spreading and proliferation by visualizing the cells on days 3 and 7 after seeding. The cells were fixed with 4% paraformaldehyde for 10 min. After fixation, the cell membrane was permeabilized with 0.1% Triton X-100 (Sigma-Aldrich Co., USA) and 1% Tween (Sigma-Aldrich Co., USA). Then the F-actin cytoskeleton was stained with phalloidin conjugated with TRITC fluorescent dye (Sigma-Aldrich Co., USA; diluted in PBS, 5 µg/mL) and the cell nuclei were stained with DAPI (Sigma-Aldrich Co., USA; diluted in PBS, 1 µg/mL), for 1 h in the dark. The cell pictures were taken under a Leica TCS SPE DM2500 confocal microscope (Leica Microsystems, Germany; obj. 20×/0.70 NA oil, 40×/1.15 NA oil). 

The cell proliferation was also evaluated by the CellTiter 96^®^ AQueous One Solution Cell Proliferation Assay (MTS, Promega Corporation, Madison, WI, USA) on days 3 and 7 after cell seeding. The samples were rinsed with PBS and were moved into fresh cell culture wells in order to avoid the influence of the cells adhered to the bottom of the wells. The assay was performed according to the manufacturer’s protocol. The formazan dye produced by the cells after 2 h of incubation was quantified by measuring the absorbance using a VersaMax ELISA Microplate Reader spectrophotometer (Molecular Devices Corporation, Sunnyvale, CA, USA). The absorbance was measured with wavelength 490 nm. The measured quantitative data were presented as the arithmetic mean ±S.D. of three independent samples for each experimental group and time interval. The statistical significance was evaluated using the analysis of variance (One Way ANOVA–Tukey method). Values of *p* ≤0.05 were considered as significant. 

## 3. Results

### 3.1. Structural Morphology and Chemical Characterization and of Pristine Hcel^®^ NaT

Both forms of Hcel^®^ NaT (P and H forms) had a fibrous morphology with fiber diameter in the microscale. The fiber diameter was characterized by fiber width and thickness (Table 1). The average fiber width was 18 µm, and the average fiber thickness was approximately 6 µm for both forms of Hcel^®^ NaT. The area of the void spaces among the fibers varied approximately from 100 to 8000 µm^2^ (Table 1).

The two prepared forms of Hcel^®^ NaT differed in molecular weight, in the degree of hydroxyl group substitution by carboxymethyl group, and in the pH of their aqueous extracts, as shown in Table 2. 

**Figure 1:**
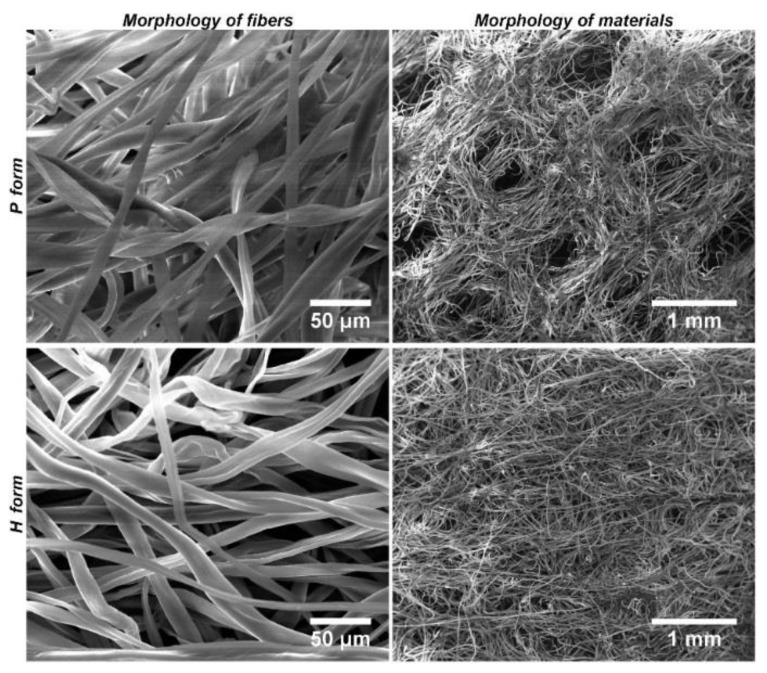
Morphology of the P form and the H form of Hcel^®^ NaT. Quanta 450 scanning electron microscope, original magnification 1000×, scale bar 50 µm; 70×, scale bar 1 mm.

Moreover, the forms of Hcel^®^ NaT differed in their structural morphology. The P form had visible pores, and the fibers were randomly assembled in a grid shape, whereas the H form had fibers homogeneously distributed over the entire surface (Figure 1).

### 3.2. Morphology of the Fibrin Structures

**Figure 2:**
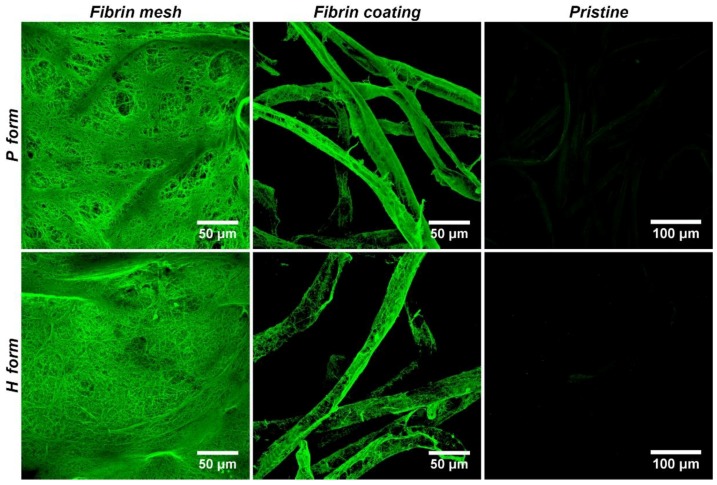
Morphology of the P form and the H form of Hcel^®^ NaT modified with a fibrin mesh or a fibrin coating. The pristine Hcel^®^ NaT of P form and H form were used to show non-specific binding of the antibodies. The fibrin was stained with a primary polyclonal rabbit antibody against fibrinogen and with a secondary antibody conjugated with Alexa 488 (green fluorescence). Leica TCS SPE DM 2500 confocal microscope, magnification 40×/1.15 NA oil or magnification 20×/0.70 NA oil.

The P and H forms of the Hcel^®^ NaT were modified with two different fibrin structures (fibrin mesh and fibrin coating). The fibrin mesh coated the material fibers, and in addition it formed a homogeneous fine mesh of nanofibers lying on the membrane fibers and spreading among them. The fibrin nanofibrous mesh homogenously filled the void spaces between membrane fibers which reduced the pore size. The fibrin coating just covered the surface of individual membrane fibers with fibrin nanofibers and did not significantly affect the area of void spaces between fibers of the membrane (Figure 2). In our previous studies performed on nanofibrous polylactide meshes, we found that the fibrin structure was stable for 7 days under cell culture conditions without cells, whereas the fibrin was gradually degraded on the membrane with cells [18,20].

### 3.3. Cell Adhesion and Proliferation on the Pristine Hcel^®^ NaT

**Figure 3:**
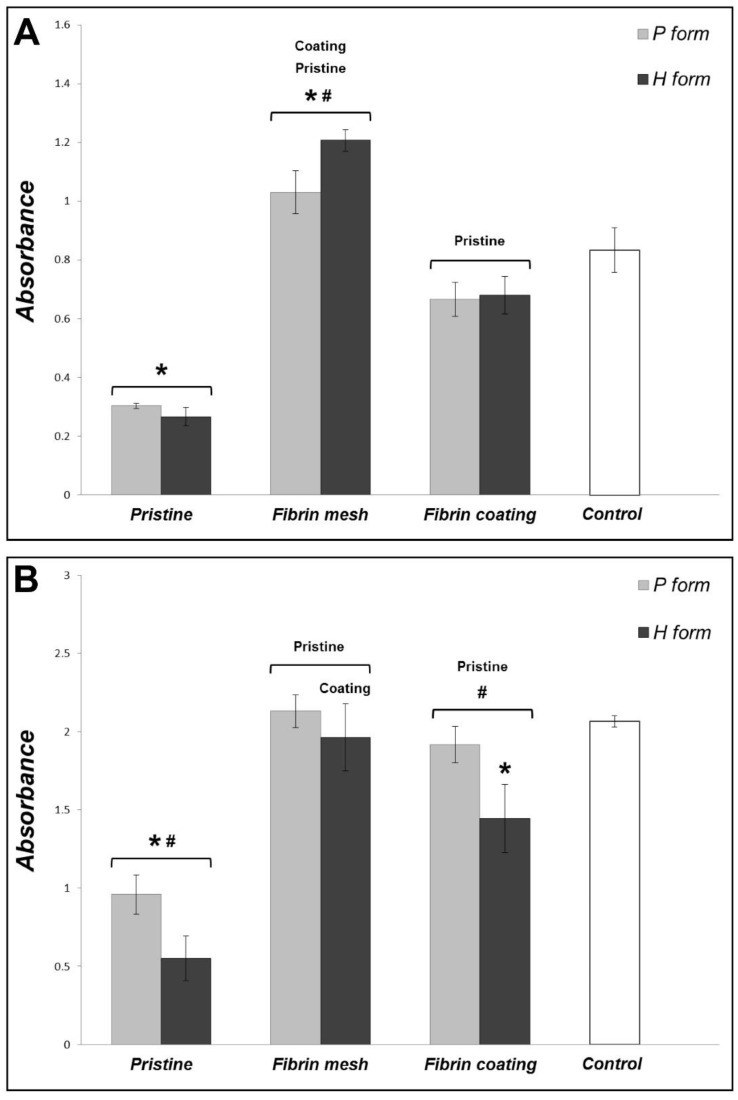
Mitochondrial activity of human dermal fibroblasts on the P form and the H form of Hcel^®^ NaT in pristine state and modified with a fibrin mesh or a fibrin coating, and on the bottoms of the control polystyrene culture wells on day 3 (A) and on day 7 (B) after cell seeding. The absorbance was calculated as the arithmetic mean ± S.D. from three independent samples for each experimental group and time interval. ANOVA, Tukey method, statistical significance (*p* ≤0.05) above the column on or under the line: * compared with the control polystyrene, ^#^ between the P form and the H form, Pristine or Coating compared with Pristine or Fibrin mesh Hcel^®^ NaT. The labels of statistical significance on the line are for both columns, the labels under the line are for a specific column.

The proliferation of human dermal fibroblasts was estimated by measuring the activity of cell mitochondrial enzymes (determined by an MTS assay). The cells did not adhere and proliferate well on either form of the pristine Hcel^®^ NaT. The cell proliferation was significantly lower on both forms of Hcel^®^ NaT on days 3 and 7 after cell seeding than on the bottoms of the control polystyrene culture wells, and this difference increased with the time of cell cultivation. We also observed slightly better cell adhesion and proliferation on the P form than on the H form, with statistical significance on day 7 (Figure 3).

**Figure 4:**
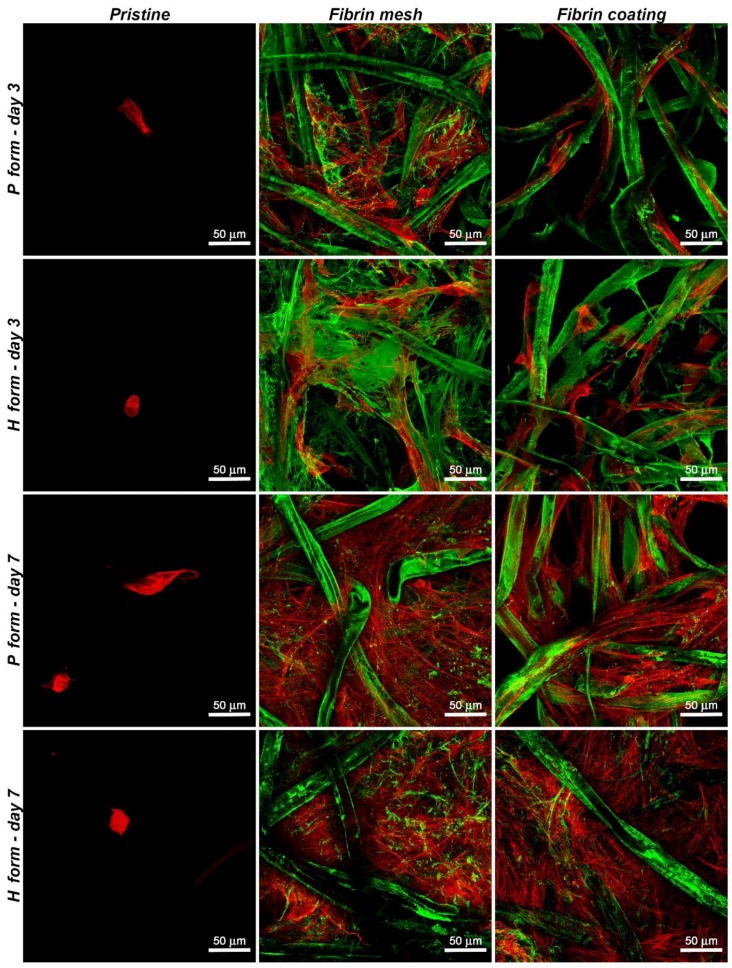
Morphology of human dermal fibroblasts on the P form and on the H form of Hcel^®^ NaT, in pristine state and modified with a fibrin mesh or a fibrin coating on day 3 or day 7 after cell seeding. The fibrin was stained with the primary and secondary antibodies mentioned above (Alexa 488, green), and the F-actin cytoskeleton was stained with phalloidin-TRITC (red). Leica TCS SPE DM 2500 confocal microscope, magnification 40×/1.15 NA oil.

Fluorescence microscopy confirmed the data obtained by the MTS assay. We observed only a small number of adhered cells on both forms of pristine Hcel^®^ NaT. The cells on the P form seemed to be elongated on the fibers. However, the cells on the H form were mostly of rounded shape, which indicates poor cell adhesion. The number of cells increased slightly on the P form on day 7 compared to day 3. However, the number of cells on the control polystyrene was much higher. On the H form, on day 7, the cells still had a rounded shape and the cell density remained similar as on day 3 (Figure 4, 1st column Pristine).

### 3.4. Cytotoxicity of Hcel^®^ NaT

**Figure 5:**
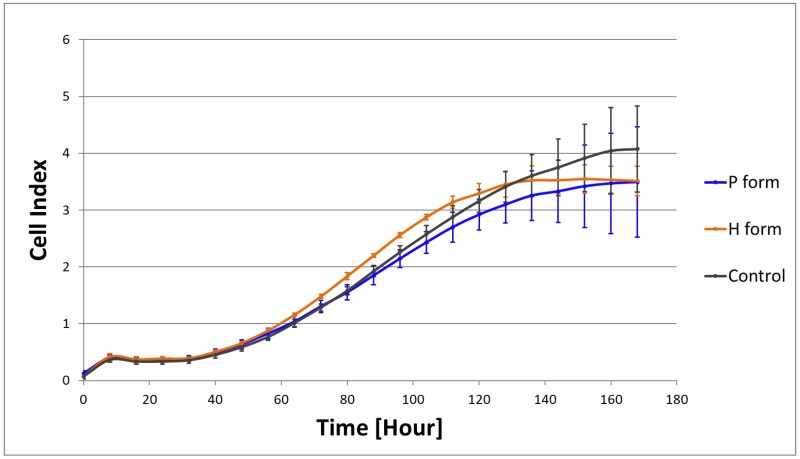
Proliferation of human dermal fibroblasts indicated by the Cell Index in extracts from the P form and the H form of Hcel^®^ NaT, and in the control culture DMEM medium. Measured by the real-time xCELLigence^®^ monitoring system for 168 h with 8-h intervals. Arithmetic mean ±S.D. from four independent samples for each type of material.

The poor cell adhesion and proliferation on the pristine Hcel^®^ NaT may have been caused by a release of cytotoxic elements from the material. To determine the potential cytotoxic effect of the material, we cultured cells in the extracts from the material samples using the real-time xCELLigence^®^ monitoring system. The cells proliferated in the material extracts similarly as in the control pure culture medium. The cell density increased with cultivation time, as indicated by the increasing Cell Index values (Figure 5).

### 3.5. Cell Adhesion and Proliferation on Fibrin-Modified Hcel^®^ NaT

We suggested that the material properties might be improved by modifying them with fibrin of two different structures (fibrin mesh and fibrin coating). The cells grew well on both fibrin structures, and their proliferation, estimated by the cell mitochondrial activity, was significantly higher than on the pristine material in all time intervals. A comparison of the cell behavior on the two different fibrin structures showed that there was higher cell proliferation on the fibrin mesh than on the fibrin coating, mainly on day 3 after seeding (Figure 3). Moreover, the cell proliferation differed slightly on the P and H forms of Hcel^®^ NaT modified with a fibrin mesh or a fibrin coating. Regarding the fibrin mesh structure, we observed significantly higher cell proliferation on the H form than on the P form on day 3. However, on day 7, the cell proliferation on the fibrin mesh tended to be higher on the P form. The fibrin coating significantly increased the cell proliferation on the P form on day 7.

The fluorescence microscopy images (Figure 4) showed that the cells adhered much better to both forms of Hcel^®^ NaT modified with fibrin structures, either the fibrin mesh or the fibrin coating, than on the unmodified materials. The cells were spread through the fibrin mesh covering and interconnecting the cellulose fibers, and tended to be polygonal in shape. By contrast, on the fibrin coating, the cells copied the cellulose fibers and remained in a spindle-like morphology. The number of adhered cells increased with the cultivation time, and the cells achieved a confluent layer on both forms of Hcel^®^ NaT modified with fibrin structures on day 7. The cells progressively degraded and reorganized the fibrin mesh and the fibrin coating during their cultivation. After 7 days of cell cultivation, the thin homogeneous mesh lying on the fibers and spreading between them was almost completely degraded, whereas the cellulose fibers remained coated with fibrin.

## 4. Discussion

Cellulose and other polysacharide materials are clinically applied to support wound healing by covering the wound, and also to release drugs and other bioactive molecules into wounds. For example, Tamer et al. (2018) prepared membranes composed of chitosan and high-molar-mass hyaluronan loaded with a gradually releasing mitochondrially targeted antioxidant–Mito Q. The addition of Mito Q antioxidant had beneficial effect on the healing process of injured rabbit and rat skin in vivo, which was due to a suppression of inflammation process caused by reactive oxygen species produced by mitochondria during insufficient oxygen uptake [23]. The application of cellulose as a direct skin tissue substitute with adhered skin cells is still relatively rare. The reason is that this application requires degradability of the cellulose-based material, or spontaneous removal of the material from the wound, in order to prevent inflammation or scar formation. Cellulose can be rendered degradable e.g., by chemical modification or by combining it with other scaffold components. For example, biodegradability has been achieved in a hydroxyethyl cellulose/poly (vinyl alcohol) blend in the form of electrospun fibrous membranes [24], in a cellulose film regenerated from *Styela clava* tunics [25], and in enzyme-digestible cellulose membranes [12]. Another option is to use non-degradable carriers, which can be self-detached from the wound after cell adhesion on the wound bed. These carriers include e.g., a polyurethane wound dressing known as HydroDerm [10], a porous dressing made of a copolymer of hydrophilic polyethylene glycol terephthalate soft segments and polybutylene terephthalate hard segments [11], electrospun mats consisting of polycaprolactone (PCL) and polyvinyl alcohol (PVA) [26], and a composite hydrogel consisting of bacterial cellulose and acrylic acid [13]. The carboxymethylcellulose scaffolds developed in our study are further potential non-degradable carriers for cell delivery into damaged skin sites.

Cellulose in its pristine state does not normally allow sufficient colonization of the material by cells, due to its inappropriate physicochemical properties. The properties of the cellulose-based scaffold can be tailored by a variety of modifications, including functionalization with specific chemical groups (e.g., –COOH), bioactive molecules (ECM proteins, growth factors, healing agents) and chemical groups, by combination with other synthetic or natural materials, by creating a nanostructured surface, by mineralization etc. [1,27]. 

In our study, we prepared a sodium carboxymethylcellulose scaffold (Hcel^®^ NaT) and we functionalized it with fibrin, a provisional ECM molecule, in order to increase its attractiveness for colonization with skin cells, and thus to stimulate better wound healing. We carried out experiments on two forms of Hcel^®^ NaT with different structural morphologies and chemical properties. The P form formed visible pores in its structure which can allow better gas and nutrient exchange during wound healing. This is one of the requirements for wound dressings that will support sufficient wound healing [28]. On the other hand, there is a potential risk of drying of the wound through the pores. Moreover, healing can take place non-homogeneously. This phenomenon was observed when the Biobrane porous wound dressing was used [29]. By contrast, the homogeneous H form can have a greater surface for potential adsorption of remedial substances, e.g., growth factors, antimicrobial agents, drugs, vitamins, hyaluronan, or fibrin [30,31,32]. 

The human dermal fibroblasts cultivated on our pristine Hcel^®^ NaT membrane showed low ability to adhere, spread and proliferate. However, the cultivation of cells in the material extracts did not confirm a cytotoxic effect of the material on the cells. For this reason, we attributed the poor cell adhesion and growth to inappropriate material surface properties. Both forms of Hcel^®^ NaT had an average fiber width and thickness in the microscale. Microscale surface roughness is often inappropriate for cell adhesion and spreading. The size of the irregularities (the Hcel^®^ NaT fibers) is similar to the spreading area of the cells. As a result, the cells cannot adhere to the material by whole their spreading area and they try to bridge the irregularities or squeeze between them [33]. Moreover, the area of void spaces among the fibers was from hundreds to thousands of square micrometers. The non-adhered round cells were about 15 µm in diameter, so it was possible for cells to fall through the void spaces in the material to the bottom of a culture well. 

The poor cell adhesion on both pristine forms of Hcel^®^ NaT could also be due to the excessively soft surface properties of the material fibers. As Hopp et al. [34] showed, fibroblasts prefer a stiffer surface to a softer surface. The Hcel^®^ NaT fibers tended to form a gel structure in the cell culture medium when the liquid was absorbed into the material. A soft gelling material surface may collapse under the traction forces generated by the adhering cells [35]. It was mentioned above that the degree of substitution of cellulose hydroxyl groups influences the water absorption into the material, resulting in gelling and dissolution of the material [5]. A higher degree of substitution of the H form led to more apparent gelling of the surface of the material fibers. In other words, the H form formed a softer and more collapsible structure than the P form. The cells therefore adhered and grew better on the P form than on the H form. This was manifested by the greater cell density on the P form in all time intervals.

We functionalized the Hcel^®^ NaT with two fibrin structures of different morphologies (a fibrin mesh or a fibrin coating) to enhance the attractiveness of the material for the adhesion and proliferation of the cells. The interactions between Hcel^®^ NaT and fibrin are based on the general principle of chemical bonds formation between reactive chemical groups of fibrinogen and carboxymethylcellulose. The fibrinogen is physically adsorbed on the carboxymethylcellulose through non-covalent strong physical interactions. These interactions involve ionic bonds between carboxyl groups of Hcel^®^ NaT and amino groups of fibrinogen, hydrogen bonds between hydroxyl groups of Hcel^®^ NaT and polypeptide backbone of fibrinogen, and hydrophobic interactions between non-polar parts of Hcel^®^ NaT (methyl groups) and non-polar amino acids in fibrinogen molecules. Both types of modifications with fibrin formed a network of fibrin nanofibers and thus altered the material surface roughness from microstructure to nanostructure that is considered as more favorable for cell adhesion and growth. The fibrin mesh formed a homogeneous thin nanostructure lying on the material surface and filling the void spaces among the cellulose fibers, whereas the fibrin coating only covered the cellulose fibers with fibrin nanofibers. The different morphologies of these two fibrin structures on Hcel^®^ NaT were caused by the different preparation methods, as described in our previous study [20]. The fibrin mesh was prepared without washing out the thrombin, while the preparation of the fibrin coating included a step in which the unbound thrombin with Tris Buffer was washed out. The activity and the concentration of thrombin bound to fibrinogen are crucial for the formation of the final fibrin network. Higher concentration of thrombin (above 1U/mL) induces the formation of thin fibrin fibers [36]. In our case, the high concentration of thrombin on the surface of the material enabled the formation of a homogeneous nanofibrous fibrin mesh. In addition, the desirable morphology, thickness and density of fibrin nanostructure can be also tuned by the concentration of fibrinogen and antithrombin III in the solution and by polymerization time [37]. In our study, we applied a solution of high concentrated fibrinogen (200 µg/mL) and antithrombin III of (0.5 U/mL) for 60 min. Under these conditions, we prepared well developed mesh of fibrin nanofibers. Similar results were obtained Riedelova-Reicheltova et al. on a glass surface [37]. 

Both fibrin structures greatly improved the cell adhesion and the subsequent proliferation. The number of adhered cells increased in time, and the cells reached an almost confluent layer on the 7th day of cultivation. As was mentioned above, fibroblasts can bind to fibrin via integrin and non-integrin adhesion receptors, which support fibroblast proliferation and growth factor secretion [15,38]. In our earlier studies, we observed a positive influence of fibrin modification of a nanofibrous polyester membrane on adhesion, proliferation and ECM protein synthesis in the case of dermal fibroblasts. The proliferating cells gradually degraded and reorganized the fibrin, and replaced it by their own ECM, mainly by collagen I and fibronectin, during cultivation [18,19].

Our results also revealed that the structure of the fibrin played an important role in cell adhesion and growth. Our previous study performed on polylactide nanofibrous membranes revealed that the fibrin mesh enhanced the cell adhesion, proliferation and synthesis of ECM proteins better than the fibrin coating [20]. In this study, the Hcel^®^ NaT modified with the fibrin mesh also provided better support for the attachment, proliferation and migration of cells than the fibrin coating. The fibrin mesh homogeneously distributed among the cellulose fibers decreased the area of void spaces between membrane fibers (pore size) and thus prevented the cells falling through the void spaces in the material on the bottom of the culture well. For this reason, there was a higher initial number of cells adhering to the material with the fibrin mesh than on the material modified with a fibrin coating. The overall surface for cell adhesion was also larger than in the case of the fibrin coating. In addition, the fine fibrin mesh formed a continuous nanoscale structure [39]. It is generally accepted that nanostructured materials better mimic the natural cell environment of the ECM, and cell propagation is more successful on these biomimetic scaffolds [39]. In addition, nanostructured materials promote the adsorption of cell adhesion-mediating molecules in an almost physiological spatial conformation, which enables binding between specific amino acid sequences in these molecules, e.g., RGD, and cell adhesion receptors, e.g., integrins [40,41]. In addition, fibroblasts were capable of remodeling a nanofibrous fibrin mesh, which facilitated fibroblast migration and proliferation, and synthesis of ECM [42]. 

Although the different properties of the P and H forms of Hcel^®^ NaT did not apparently affect the formation of fibrin structures, the cell proliferation differed slightly on these two forms modified with fibrin structures. Whereas in an earlier cell cultivation interval (on day 3) the cells proliferated better on the fibrin-modified H form (in the case of the fibrin mesh), in a later cultivation interval (on day 7), the cell proliferation was higher on the P form (particularly on the fibrin coating). It can be supposed that the H form without visible pores enables the formation of a more homogenous fibrin mesh among cellulose fibers and ensures better support for the cells than the P form in earlier cultivation intervals. However, during cell cultivation, we observed gradual degradation and remodeling of the fibrin structures. After 7 days, the fibrin, mainly the fibrin mesh, was almost degraded, and the Hcel^®^ NaT properties started to have a predominant influence on the cell behavior. As has been discussed above, the H form had less appropriate properties for cell adhesion, due to its greater tendency to form a gel structure. For this reason, in later cell cultivation intervals, the cell tended to proliferate better on the fibrin-modified P form, which has more favorable properties for cell living compared to the H form.

Our newly developed carboxymethylcellulose scaffolds coated with fibrin could be used as carriers for transferring skin cells into skin wounds. The fibrin coating would increase the cell adhesion and proliferation, and this would result in a considerably higher number of cells delivered into the wounds. Low efficiency of cell delivery was a problem with HydroDerm polyurethane wound dressings, where the keratinocytes grew at approximately 15% of the rate observed in cells cultivated on the tissue culture plastic [10]. Similarly, in polycaprolactone/polyvinyl alcohol electrospun scaffolds, the attachment, viability and proliferation of human fibroblasts was markedly improved by coating the scaffolds with fibronectin, especially when the scaffolds were loaded with an antimicrobial silver sulfadiazine agent [26].

After degradation of the fibrin coating by the cells, our cellulose scaffolds would become less attractive than the wound bed for cell adhesion, and therefore a spontaneous release and migration of cells from these scaffolds could be expected. The scaffolds could then be freely removed from the wound. In other words, our fibrin-modified cellulose-based scaffolds could serve as temporary carriers for in vitro expansion and subsequent delivery of cells into skin wounds. A similar phenomenon was observed in human keratinocytes cultured on poly(2-hydroxyethyl methacrylate) sheets, which were applied clinically in the treatment of severe burns [43].

## 5. Conclusions

In this study, we have compared the behavior of human dermal fibroblasts in cultures on a porous (P) form and a homogeneous (H) form of carboxymethylcellulose wound dressings (Hcel^®^ NaT), which differed in structural morphology and physicochemical properties. Both forms of pristine Hcel^®^ NaT showed low ability to support cell adhesion and proliferation, probably due to inappropriate physicochemical properties and inappropriate morphology of the material surface. The cell proliferation was slightly increased on the P form of Hcel^®^ NaT. However, the results showed that the cell adhesion and proliferation were greatly improved by modification of Hcel^®^ NaT by fibrin in the form of a mesh and in the form of a coating. Both fibrin structures enhanced the colonization of our wound dressing with the cells. Moreover, the fibrin mesh, resembling the natural ECM, provided better support than the fibrin coating for the adhesion and proliferation of the cells. The Hcel^®^ NaT wound dressing functionalized with fibrin, especially in the form of a mesh filling the void spaces among the cellulose fibers, is a promising tool for faster wound healing with better aesthetic outcomes. Fibrin ensures good cell adhesion and spreading, and can support the migration and the subsequent proliferation of cells in a wound.

## Figures and Tables

**Table 1:** Morphological parameters of the P and H forms of Hcel^®^ NaT.

Form of Hcel^®^ NaT	Mean Fiber Width ± S.D. (µm)	Mean Fiber Thickness ± S.D. (µm)	Range of Area of Void Spaces (µm^2^)
P form	18.1 ± 3.6	5.7 ± 1.7	104–3390
H form	18.0 ± 2.0	6.1 ± 1.3	86–8732

**Table 2:** Chemical characterization of the P and H forms of Hcel^®^ NaT.

Form of Hcel^®^ NaT	Molecular Weight (kDa)	Degree of Substitution	pH of an Aqueous Extract
P form	262	0.120	8.60
H form	251	0.194	7.07
